# Comparison of the pathogenic potential of highly pathogenic avian influenza (HPAI) H5N6, and H5N8 viruses isolated in South Korea during the 2016–2017 winter season

**DOI:** 10.1038/s41426-018-0029-x

**Published:** 2018-03-14

**Authors:** Hyeok-il Kwon, Eun-Ha Kim, Young-il Kim, Su-Jin Park, Young-Jae Si, In-Won Lee, Hiep Dinh Nguyen, Kwang Min Yu, Min-Ah Yu, Ju Hwan Jung, Won-Suk Choi, Jin Jung Kwon, Su Jeong Ahn, Yun Hee Baek, Dam Van Lai, Ok-Jun Lee, Si-Wook Kim, Min-Suk Song, Sun-Woo Yoon, Chul-Joong Kim, Richard J. Webby, In-Pil Mo, Young Ki Choi

**Affiliations:** 1 College of Medicine and Medical Research Institute, Chungbuk National University, Cheongju, KS001 Korea; 2 College of Veterinary Medicine, Chungbuk National University, Cheongju, KS001 Korea; 3 Korea Research Institute of Bioscience and Biotechnology, DaeJeon, KS015 Korea; 4 College of Veterinary Medicine, Chungnam National University, DaeJeon, KS015 Korea; 5 Department of Infectious Diseases, St. Jude Children’s Research Hospital, Memphis, TN TN38105 USA

## Abstract

Highly pathogenic avian influenza (HPAI) A(H5N6) and A(H5N8) virus infections resulted in the culling of more than 37 million poultry in the Republic of Korea during the 2016/17 winter season. Here we characterize two representative viruses, A/Environment/Korea/W541/2016 [Em/W541(H5N6)] and A/Common Teal/Korea/W555/2017 [CT/W555(H5N8)], and evaluate their zoonotic potential in various animal models. Both Em/W541(H5N6) and CT /W555(H5N8) are novel reassortants derived from various gene pools of wild bird viruses present in migratory waterfowl arising from eastern China. Despite strong preferential binding to avian virus–type receptors, the viruses were able to grow in human respiratory tract tissues. Em/W541(H5N6) was found to be highly pathogenic in both chickens and ducks, while CT/W555(H5N8) caused lethal infections in chickens but did not induce remarkable clinical illness in ducks. In mice, both viruses appeared to be moderately pathogenic and displayed limited tissue tropism relative to HPAI H5N1 viruses. Em/W541(H5N6) replicated to moderate levels in the upper respiratory tract of ferrets and was detected in the lungs, brain, spleen, liver, and colon. Unexpectedly, two of three ferrets in direct contact with Em/W541(H5N6)-infected animals shed virus and seroconverted at 14 dpi. CT/W555(H5N8) was less pathogenic than the H5N6 virus in ferrets and no transmission was detected. Given the co-circulation of different, phenotypically distinct, subtypes of HPAI H5Nx viruses for the first time in South Korea, detailed virologic investigations are imperative given the capacity of these viruses to evolve and cause human infections.

## Introduction

Highly pathogenic avian influenza (HPAI) H5 viruses have been continuously isolated from wild birds and domestic poultry since the first detection of A/Goose/Guangdong/1/1996 (Gs/GD/1996, H5N1) in poultry in 1996 in Southeast Asia^1^. HPAI H5 viruses can cause high mortality in poultry resulting in serious economic losses for the industry. Beginning in 2008, the H5N1 viruses evolved into novel reassortant H5Nx viruses of different neuraminidase subtypes^2^. Most of the H5Nx viruses, including H5N2, H5N6 and H5N8, recently circulating worldwide are clustered into a sublineage of clade 2.3.4 or 2.3.4.4^3^. Of these, the clade 2.3.4.4 H5N8 viruses were the first reported in Korea in 2014^4^. These viruses subsequently spread to East Asia, North America and Europe^5^. In North America the H5N8 viruses reassorted with wild-bird influenza viruses and transiently infected domestic turkeys and chickens^5,6^.

In addition to the H5N8 viruses, a clade 2.3.4.4 H5N6 virus emerged in China in 2013 and spread to Lao PDR and Vietnam in 2014–2015. The H5N6 virus has caused high mortality in domestic poultry and now appears to be well established in Lao PDR, Vietnam, and Mainland China^7,8^. Although, similarly to H5N8, the H5N6 HA belongs to clade 2.3.4.4, it appears to be more virulent and has caused 17 human infections with 7 fatal cases in China as of mid-2017^9^.

In the Republic of Korea, the H5N6 virus was first found in late October of 2016 in fecal specimens from migratory wild birds and went on to cause poultry outbreaks in mid-November 2016^10^. Outbreaks in domestic poultry were spatially and temporarily associated with die-offs of wild birds, leading to speculation that migratory waterfowl were the source of infection^9^. During the 2016/17 outbreak, additional novel H5N8 viruses were isolated from fecal specimens of wild birds in the Gyeonggi Province of central South Korea and subsequently caused devastating outbreaks in domestic poultry^11^. Genetic characterization of EM/W541 (H5N6) and CT /W555 (H5N8) showed that both are novel viral reassortants of clade 2.3.4.4 HPAI H5Nx and co-circulating low pathogenic avian influenza (LPAI) viruses. Briefly, EM/W541 (H5N6) is a reassortant with PB2, HA, M and NS genes from A/Dk/Guangzhou/41227/2014 (H5N6)-like virus, NP and NA genes from A/Ck/Shenzhen/1061/2013 (H5N6)-like virus, PB1 gene from A/Dk/Guangdong/S4040/2011 (H4N2)-like virus, and PA gene from A/Dk/Mongolia/520/2015 (H1N1)-like virus^9^. Further, CT/W555/H5N8 is a reassortant with HA and NA genes from A/DK/EasternChina/S1109/2014 (H5N8)-like virus, PB2, NP, M and NS genes from A/EM/Korea/W437/2012 (H7N7)-like virus, PB1 gene from A/DK/Mongolia/709/2015 (H10N7)-like virus, and PA gene from A/RS/Mongolia/1-26/2007 (H3N8)-like virus^11^.

The continued presence of the H5N6 and H5N8 viruses in poultry and wild birds has raised questions as to their immediate public health threat. Considering this and the co-circulation of two different HPAI subtypes for the first time in South Korea, we undertook a detailed assessment of the risk posed by H5N6 and H5N8 viruses.

## Results

### Genetic characterization

During this study, six H5N6 and five H5N8 viruses were isolated from specimens collected from migratory birds. Full-length sequencing results showed that each subtype of viruses have 98 to 100% homology with each other. Further, comparison with a report by Song B. M. et al. revealed that our wild bird isolates in South Korea are closely associated with the dominant H5N6 and H5N8 subtypes in domestic poultry^12^. Therefore, we selected the A/Environment/Korea/W541/2016 and A/Common Teal/Korea/W555/2017(H5N8) strains as representatives of H5N6 and H5N8 subtypes for further study. These will be referred to as EM/W541 (H5N6) and CT/W555 (H5N8) from here on. Further, genetic characterization revealed that the 2016/17 Korean H5N8 viruses were novel reassortants between group B H5N8 viruses and low pathogenic avian influenza viruses circulating in South Eastern Asia, rather than descendants of 2014/2015 Korean H5N8 viruses^4,13^.

The deduced amino acid (aa) sequences of the HA proteins of the isolated viruses revealed a series of polybasic amino acids at the HA cleavage site (RERRRKR/G in H5N6 and REKRRKR/G in H5N8), a common characteristic of avian influenza viruses that are highly pathogenic in chickens^14,15^. Further, all Korean HPAI H5N6 and H5N8 isolates maintain a glutamine residue at position 226 (H3 numbering) and a glycine residue at position 228, suggestive of preferential binding to sialic acid receptors joined to sugar chains through an α-2,3 linkage as is typical for influenza viruses of avian species^16^. However, all H5N6 viruses have a one amino acid deletion (amino acid 133 of HA1) relative to H5N8 HA genes, which is commonly found with H5N6 viruses that cause human infections^17^. This position is associated with the alteration of HA receptor binding specificity and deletion of this amino acid confers an increased α-2,6 receptor preference of H5N1 viruses (Supplementary Table S1)^18,19^. In addition, all of the H5N6 viruses had the characteristic 20 amino acid NA stalk deletion (site 49 to 68), although no amino acid substitution associated with resistance to NA inhibitors was observed. Em/W541 (H5N6) and CT/W555 (H5N8) possessed glutamic acid (E) and aspartic acid (D) instead of lysine (K) and asparagine (N) at positions 627 and 701, respectively, in PB2, which are well-known virulence markers among HPAI H5 and H7 viruses^20^. Both isolates had functional PB1-F2 proteins which have been shown to impact host defense mechanisms and in turn enhance pathogenicity in vivo^21,22^. The amino acid sequence of the NS1 protein revealed that H5N6 isolates had a five-residue deletion at positions 80 to 84. This deletion has been observed in isolates from poultry in Hong Kong since 2001^23^. EM/W541 (H5N6) bears aspartic acid in place of glutamic acid at position 92 of the non-structural (NS) 1 protein compared with CT/W555 (H5N8), which is responsible for attenuating anti-viral host interferon responses. Moreover, the C-terminal PDZ-binding motifs were ESEV and GSEV for EM/W541 and CT/W555, respectably, which is typical for avian viruses and confers a severe disease phenotype in mice (Supplementary Table S1). Single nucleotide polymorphism (SNP) analysis revealed that the deletion of residue 133 in HA preferentially occurred in recently isolated human H5N6 viruses (70% vs 3.1% in human and avian isolates, respectively) (Supplementary Table S2).

### Biological properties of Em/W541 (H5N6) and CT/W555 (H5N8)

**Fig. 1:**
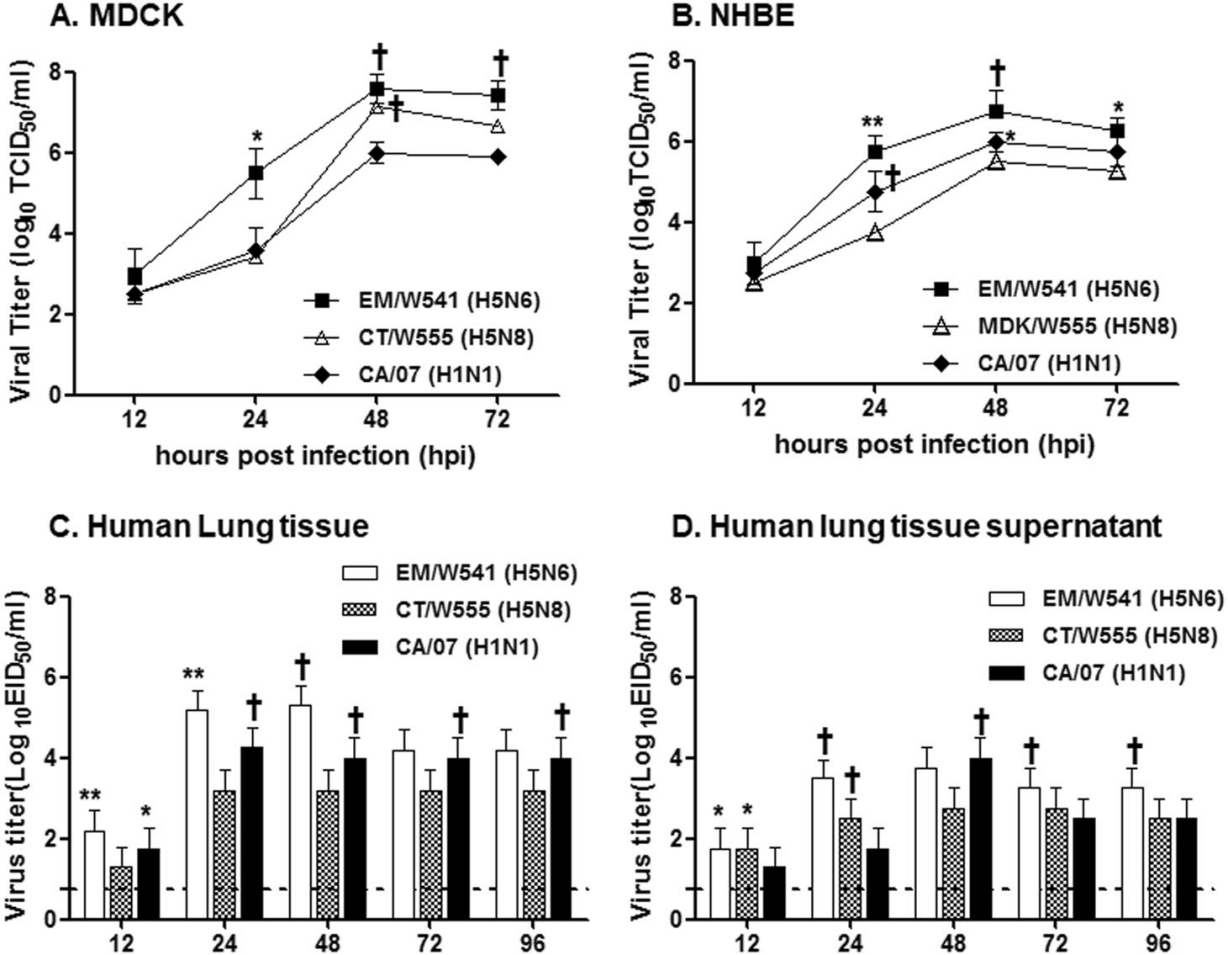
Growth kinetics and attachment of viruses in human lung tissues ex vivo. Replication of two Em/W541(H5N6) and CT/W555(H5N8) viruses were monitored in Madin-Darby Canine Kidney (MDCK) cells (**a**), Normal Human Bronchial Epithelial (NHBE) cells (**b**), Human lung tissue explants and corresponding culture supernatants (**c** and **d**, respectively) starting at 12 and 24 hpi intervals thereafter. Growth kinetics were compared to those of the control 2009 pandemic CA/07(H1N1) virus. The titers shown are means ± SD from three independently performed experiments. (*p < 0.05, **p < 0.01, ^†^*p* < 0.001)

To examine the growth properties of the Em/W541 (H5N6) and CT/W555 (H5N8) viruses *in vitro*, each virus was examined for viral growth kinetics in MDCK and human bronchial epithelial (NHBE) cells. The prototype pH1N1 virus A/California/7/2009 (CA/07, H1N1), which is known to grow efficiently in human cell lines, was included as a positive control. In MDCK cells, the replication of Em/W541 (H5N6) was higher than that of CT/W555 (H5N8) at all time points, with the exception of 48 hpi where they were comparable with both being markedly higher than that of CA/07 (H1N1) (peak titers of 6.5–8.0 log_10_TCID_50_/ml) (Fig. 1a).

In primary NHBE cells, the Em/W541 (H5N6) and CA/07 (H1N1) viruses exhibited relatively rapid growth from 24 hpi on, reaching similar titers at 72 hpi. CT/W555 (H5N8) showed delayed growth properties and had viral titers more than 10 times lower than Em/W541 (H5N6) at 48 hpi (6.75log_10_TCID_50_/ml vs 5.5log_10_TCID_50_/ml). These results show that Em/W541 (H5N6) exhibits a higher growth rate than CT/W555 (H5N8) in mammalian cell lines (Fig. 1b).

To model human respiratory tract infections, we inoculated freshly biopsied human lung tissue samples, in which the lack of influenza virus infection had been confirmed by RT-PCR, with each virus. CA/07 (H1N1) efficiently replicated in the lung tissues reaching titers of up to 4.25 log_10_EID_50_/ml at 48 hpi. Although CT/W555 (H5N8) grew to only low titers (3.2 log_10_EID_50_/ml), Em/W541 (H5N6) titers were as high as 5.3 log_10_EID_50_/ml, (*P* < 0.05 c.f. CT/W555 (H5N8)) in both lung tissues and tissue supernatant (Fig. 1c, d). Collectively, Em/W541 (H5N6) had the ability to replicate in human lung tissues in a manner equivalent to the seasonal H1N1 virus implying that humans may be more highly susceptible to this virus than to CT/W555 (H5N8).

Antiviral compounds represent the first line of therapeutic and prophylactic defense against an emerging influenza virus. Assessment of the 50% inhibitory concentration (IC_50_) of oseltamivir, zanamivir, peramivir, and laninamivir against egg-grown virus stocks revealed that Em/W541 (H5N6) and CT/W555(H5N8) are sensitive to this panel of neuraminidase inhibitors (Supplementary Table S3). This correlates with the absence of drug-resistance markers in the NA of these viruses.

### Virus replication in experimentally inoculated chickens and ducks

**Fig. 2:**
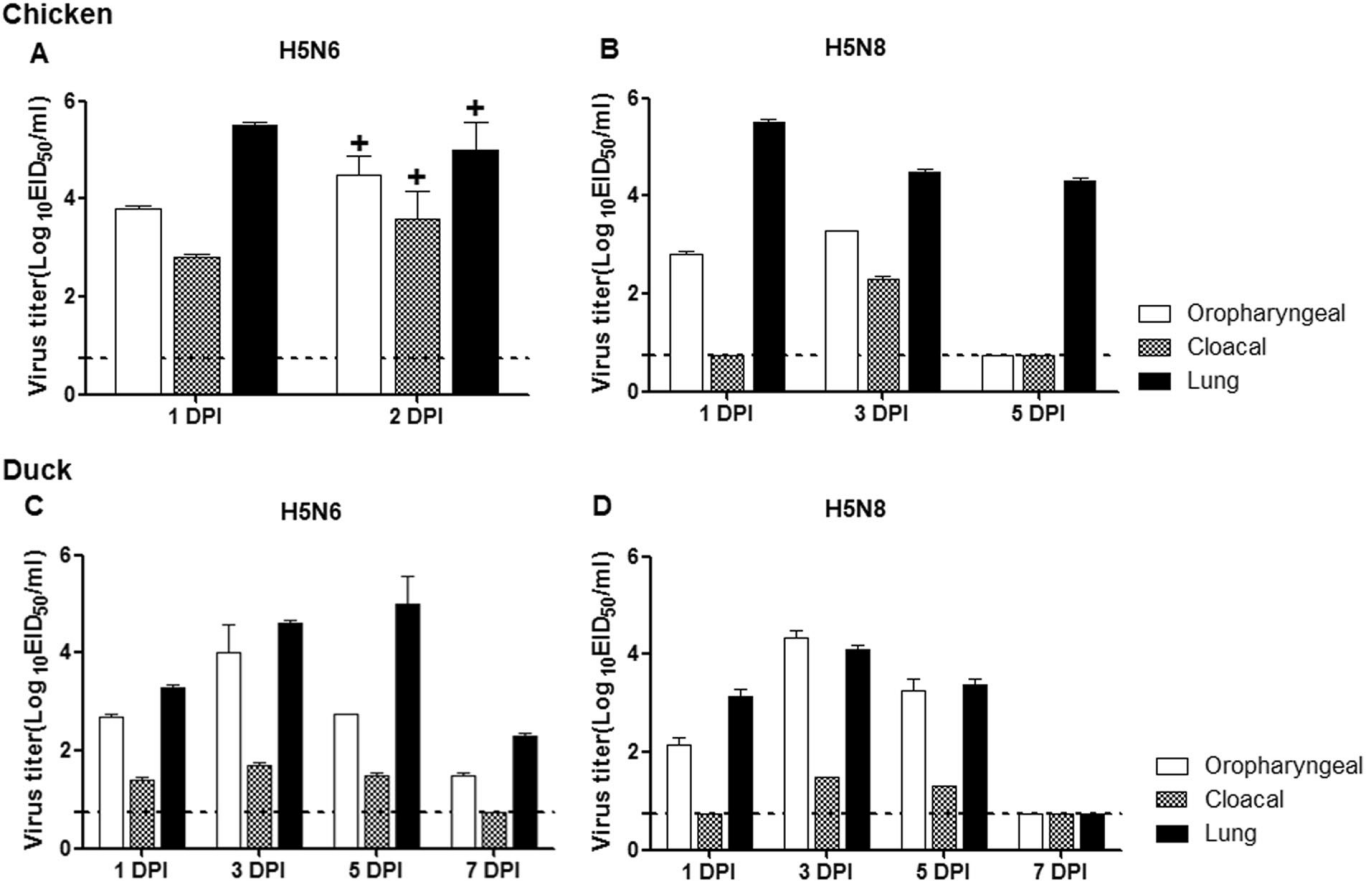
Replication of Em/W541(H5N6) and CT/W555(H5N8) in avian models. Virus replication was examined in chickens and ducks that had been experimentally inoculated via the intranasal route with 10^6^ EID_50_/ml of each virus. Oropharyngeal swabs, cloacal swab, and lung titers of chickens are shown for Em/W541(H5N6) (**a**) and CT/W555(H5N8) (**b**). Oropharyngeal swabs, cloacal swabs, and lung titers from ducks are shown for Em/W541(H5N6) (**c**) and CT/W555(H5N8) (**d**). Mean viral titers (log_10_ EID_50_/ml) are shown for each group of birds. The limit of virus detection was 0.7 log_10_ EID_50_/ml

Both Em/W541 (H5N6) and CT/W555 (H5N8) were classified as HPAI in chickens where the mean death time (MDT) was 36 and 60 h and the intravenous pathogenicity index (IVPI) was 2.66 and 2.94, respectively, as calculated according to the World Organization for Animal Health (OIE) standards^9^(Table 1). To further investigate the growth properties of these viruses in chickens, groups of nine 5-week-old chickens were oronasally inoculated with 6.0 log_10_ EID_50_/0.5 ml of virus. Oropharyngeal (OP) and cloacal (CL) swab samples were collected daily and tissue samples including lung, brain, liver, spleen, kidney, heart, intestine, and colon were also harvested at 1 and 2 dpi. Unlike CT/W555 (H5N8), Em/W541 (H5N6) virus-infected chickens died at 2 dpi limiting the number of samples collected. All OP and CL swabs from the infected chickens were positive for virus with the peak of virus detection occurring at 2 dpi for both OP swabs (4.2–5.2 log_10_ EID_50_/0.1 ml) and CL swabs (3.2–4.2 log_10_ EID_50_/0.1 ml) (Fig. 2a). In addition, Em/W541 (H5N6) was detected in all tissue samples tested including lung, brain, liver, spleen, kidney, heart, intestine, and colon and exhibited relatively high replication in the lungs (ranging from 5.0–5.5 log_10_ EID_50_/0.1 ml) and colon (ranging from 4.5–4.75 log_10_ EID_50_/0.1 ml) compared to the other organs (ranging from 2.0–3.5 log_10_ EID_50_/0.1 ml) (Table 2). Similarly, CT/W555 (H5N8) was also detected in all tissue samples although higher levels of virus replication occurred in the lung, kidney, heart, and colon (ranging from 4.5–5.5 log_10_ EID_50_/0.1 ml) than in brain, spleen, or liver samples at 1 dpi (ranging from 1.8–3.8 log_10_ EID_50_/0.1 ml) (Table 2).

**Table 1:** Biological properties of HPAI H5 viruses

Virus Name	H5 Clade	Biological characteristics						Transmission		Reference
		EID_50_	TCID_50_	Ck LD_50_	Dk LD_50_	IVPI	MLD_50_	Chicken	Duck	
Em/W541 (H5N6)	2.3.4.4	9.2	7.8	3.2	5.5	2.7	5.5	6/6	6/6	[9]
CT/W555 (H5N8)	2.3.4.4	8.9	6.5	4.7	≥6.0	2.9	5.2	6/6	6/6	[11]
MDk/W452 (H5N8)	2.3.4.4	8.9	6.9	2.5	≥6.0	3	5.8	6/6	6/6	[14]
BDk/Gochang1 (H5N8) ^*^	2.3.4.6	N/A	N/A	N/A	N/A	3^*^	N/A	N/A	N/A	[4]

All values are expressed as log_10_: EID_50_, 50% egg infectious dose; TCID_50_, 50% tissue culture infectious dose; Ck LD_50_, 50% lethal dose in chickens; Dk LD_50_, 50% lethal dose in ducks; IVPI, intravenous pathogenic index determined in chickens; MLD_50_, 50% lethal dose in mice

*N/A* not applicable

^*^As reported by Lee et al. (2014)

**Table 2:** Viral tissue titers in experimentally inoculated animals

Tissue^a^	Em/W541(H5N6)								CT/W555(H5N8)							
	Chicken		Duck		Mice		Ferret		Chicken		Duck		Mice		Ferret	
	1d^b^	2d	3d	5d	3d	5d	3d	5d	3d	5d	3d	5d	3d	5d	3d	5d
Lung	5.5 ± 0.1^c^	5.0 ± 0.2	4.6 ± 0.4	5.0 ± 0.5	4.2 ± 0.3	5.2 ± 0.3	6.0 ± 0.2	5.5 ± 0.3	5.5 ± 0.5^b^	4.5 ± 0.2	4.2 ± 0.2	3.5 ± 0.3	3.5 ± 0.3	4.3 ± 0.2	3.5 ± 0.3	3.0 ± 0.2
Brain	2.5 ± 0.4	2.0 ± 0.2	3.8 ± 0.1	3.2 ± 0.4	–	–	3.2 ± 0.1	1.2 ± 0.4	3.3 ± 0.3	–	–	–	0.8 ± 0.5	1.8 ± 0.3	1.0 ± 0.3	–
Kidney	2.5 ± 0.3	3.0 ± 0.4	2.8 ± 0.4	4.5 ± 0.1	1.0 ± 0.1	–	–	–	5.3 ± 0.3	3.3 ± 0.2	4.3 ± 0.2	3.3 ± 0.1	1.2 ± 0.5	1.0 ± 0.5	–	–
Spleen	2.5 ± 0.2	2.3 ± 0.3	1.7 ± 0.4	–	2.7 ± 0.5	–	1.0 ± 0.2	2.0 ± 0.1	3.8 ± 0.4	–	3.5 ± 0.3	1.5 ± 0.2	1.5 ± 0.1		–	–
Heart	3.5 ± 0.3	2.5 ± 0.1	4.4 ± 0.4	5.4 ± 0.4	4.2 ± 0.2	2.7 ± 0.5	–	–	5.5 ± 0.3	3.5 ± 0.1	3.8 ± 0.2	1.8 ± 0.3	1.2 ± 0.2	1.2 ± 0.3	–	–
Liver	3.0 ± 0.2	2.8 ± 0.5	1.5 ± 0.1	–	2.2 ± 0.4	–	–	–	1.8^d^	–	4.2 ± 0.2	2.7 ± 0.3	1.2 ± 0.3	1.5 ± 0.2	1.0 ± 0.2	1.0 ± 0.2
Colon	4.5 ± 0.1	4.75 ± 0.2	2.1 ± 0.4	2.7 ± 0.2	–	–	–	–	4.5 ± 0.3	3.75 ± 0.3	2.5 ± 0.1	1.3 ± 0.3	0.8 ± 0.4	1.5 ± 0.3	1.3 ± 0.3	1.0 ± 0.5

Dashed lines indicate negative for virus detection (lower limit = 0.7 log_10_EID_50_/g)

^a^Results were obtained from three animals per group at each time-point and are expressed as log_10_ EID_50_/g tissue

^b^Days post infection

^c^Standard deviation titers

^d^Positive detection in one

We next tested whether Em/W541 (H5N6) and CT/W555 (H5N8) had different pathogenicity in ducks. In the case of Em/W541 H5N6-infected ducks, the virus replicated robustly with peak titers in lungs (5.0 log_10_ EID_50_/0.1 ml), brains (3.8 log_10_ EID_50_/0.1 ml), kidneys (4.5 log_10_ EID_50_/0.1 ml), hearts (5.4 log_10_ EID_50_/0.1 ml), and colons (2.7 log_10_ EID_50_/0.1 ml) by 5 dpi with no evidence of virus in the spleen or liver (Fig. 2c and Table 2). Although CT/W555 (H5N8) was recovered from all tissues examined except brain, the peak titers were lower than those of Em/W541 H5N6.

To obtain more granularity in the nature of the infection with Em/W541 (H5N6) and CT/W555 (H5N8) we detected levels of antigen in various tissues post infection using immunostaining. Antigen-positive cells were frequently detected in lungs of chickens and ducks infected with both viruses. The levels of antigen detected in CT/W555 (H5N8)-infected chickens were substantially attenuated and the infection appeared to be less invasive than seen in the Em/W541 (H5N6)-infected chickens. Further, CT/W555 (H5N8) viral antigen was rarely detected in the kidney, pancreas, or liver of infected ducks, but was readily detectable in spleen and thymus (Supplementary Fig. S1 and Supplementary Fig. S3). For Em/W541 (H5N6), viral antigen was readily detected in thymus, kidney, pancreas, and spleen of infected ducks.

The results demonstrate that Em/W541 (H5N6) replicates to a high titer in various organs and has the potential to cause high mortality in both chicken and ducks. CT/W555 (H5N8) showed high pathogenic potentials only in chickens, but not in ducks which exhibited only moderate virus replication in extrapulmonary tissues.

### Pathogenicity of Em/W541 (H5N6) and CT/W555 (H5N8) in mice

To evaluate the pathogenic potential of Em/W541 (H5N6) and CT/W555 (H5N8) in mammals, 5-week-old female BALB/c mice were infected with each virus. Groups of mice (*n* = 6) were inoculated with the 10-fold serially diluted viruses (ranging from 10^1^ to 10^6^ EID_50_) and were monitored daily for 14 days for weight loss and survival. No significant clinical signs were observed in mice infected with less than 10^4^ EID_50_ of virus while mice infected with more than 10^5^ EID_50_ showed influenza-like symptoms, such as ruffled fur, depression, and severe weight loss. Virulence levels in mice can be categorized as low (MLD_50_ > 6.5 log_10_ EID_50_), medium (3 log_10_ EID_50_ < MLD_50_ ≤ 6.5 log_10_ EID_50_), or high (MLD_50_ ≤ 3 log_10_ EID_50_) pathogenicity^24^. The MLD_50_ of Em/W541 (H5N6) and CT/W555 (H5N8) were 5.5 and 5.23 log_10_ EID_50_, respectively, and thus, both viruses were considered to be of moderate-pathogenicity in mice. To determine the ability of these viruses to replicate in various tissues, we collected organs including brain, lung, liver, spleen, and kidney from three mice inoculated with 5.0 log_10_ EID_50_ of each virus at 3 and 5 dpi and titered the amount of virus present (Table 2). Em/W541 (H5N6) replicated well in the lungs of mice, with virus titers ranging from 4.2 to 5.2 log_10_ EID_50_ to 5 dpi. Further, Em/W541 (H5N6) was recovered from the heart (titers up to 4.2 log_10_ EID_50_/0.1 ml), kidney, liver, and spleen (1.0 log_10_ EID_50_/0.1 ml, 2.2 log_10_ EID_50_/0.1 ml, and 2.7 log_10_EID_50_/0.1 ml, respectively), but not from brains or colons, at 3 dpi. CT/W555 (H5N8) was broadly detected in tissue samples including lung (titers up to 4.3 log_10_ EID_50_/0.1 ml at 5 dpi), brain (ranging from 0.8–1.8 log_10_ EID_50_/0.1 ml), kidney (ranging from 1.0–1.2 log_10_ EID_50_/0.1 ml), heart (peak titer up to 1.2 log_10_ EID_50_/0.1 ml), liver (ranging from 1.2–1.5 log_10_ EID_50_/0.1 ml), and colon (ranging from 0.8–1.5 log_10_ EID_50_/0.1 ml) at both 3 and 5 dpi (Table 2).

**Fig. 3:**
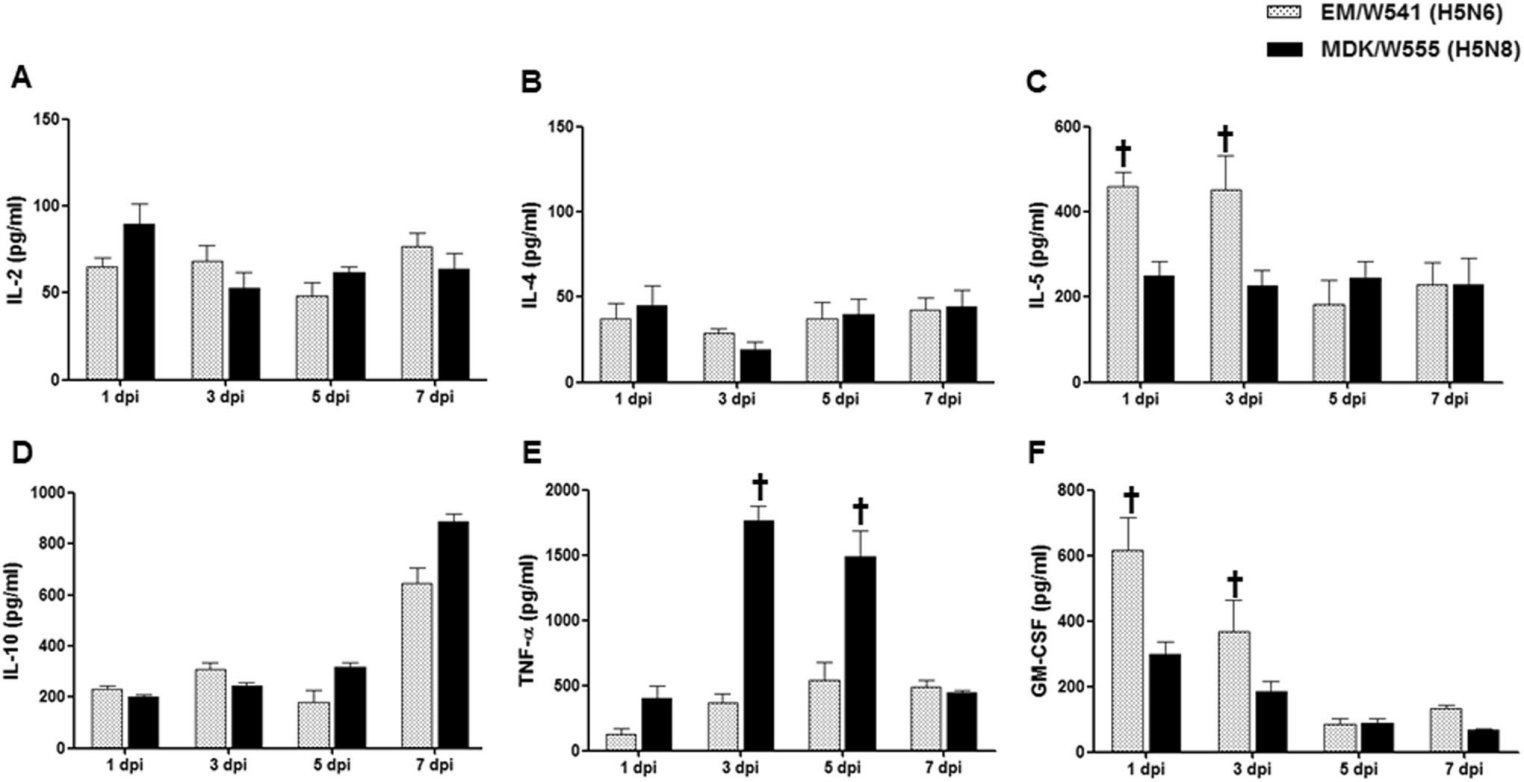
Cytokine and chemokine responses in the lungs of infected mice. Concentrations of various cytokines/chemokines in BAL fluid from mice at 1, 3, 5, and 7 dpi were measured by protein analysis with the Luminex™ Instrumentation Systems multiplex array reader (Bio-Plex Workstation from Bio-Rad Laboratories). The values shown are means ± SD (error bars) from BAL fluid of three mice per time point tested. (*p < 0.05)

**Fig. 4:**
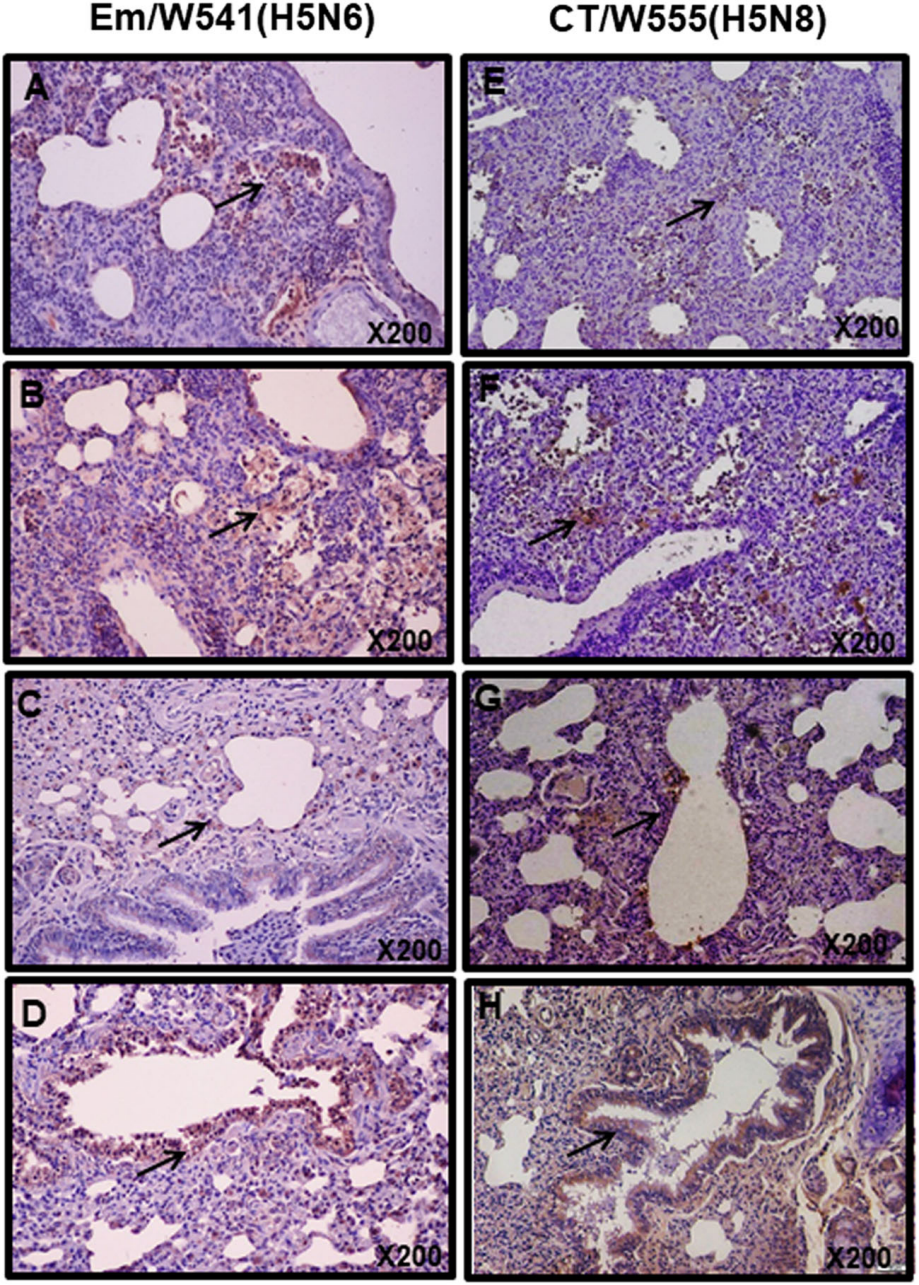
Histopathology and immunohistochemical staining for influenza virus antigen in mouse (**a**, **b**, **e**, and **f**) and ferret (**c**, **d**, **g**, and **h**) lungs at 3 (**a**, **c**, **e**, and **g**) and 5 dpi (**b**, **d**, **f**, and **h**) with HPAI Em/W541(H5N6) and CT/W555(H5N8).In the lung, viral antigens are widely presented in the alveolar septum which are mildly thickened with increased cellularity of inflammatory cells and also necrotic cell debris are presented in the lumen (arrows in **a**, **b**, **e**, and **f**)

To compare the cytokine responses in mice infected with the H5N6 and H5N8 viruses, expression analysis of Th1-associated cytokines (IL-2, IL-12, and IFN-γ), Th2-associated cytokines (IL-4, IL-5, and IL-10), the pro-inflammatory cytokine TNF-α, and the chemokine GM-CSF were assessed post-infection in BALF. Expression of IL-2, IL-4, and IL-10 were upregulated during the infection of both viruses, although there were no significant differences between viruses (Fig. 3a, b, d). In contrast, IL-5 and GM-CSF expression levels and were significantly increased in Em/W541 (H5N6)-infected mice at 1 and 3 dpi compared with CT/W555 (H5N8)-infected mice (*P* < 0.05) (Fig. 3c, f). Conversely, TNF-α was more highly expressed in the lungs of CT/W555 (H5N8)-infected mice at 3 and 5 dpi than in Em/W541 (H5N6)-infected mice (Fig. 3e). Expression of Th1 cytokines (IL-12 and IFN-γ) were below the level of detection (data not shown). These results suggest that the differential expression of IL-5, TNF-α, and GM-CSF might be associated with differences in the pathogenesis of each virus. Histologically, H5N6 and H5N8 virus infected cells were broadly detected in the lungs at 3 and 5 dpi indicating that the viruses could replicate in mice without prior adaptation (Fig. 4).

### Viral replication and pathogenesis in ferrets

**Fig. 5:**
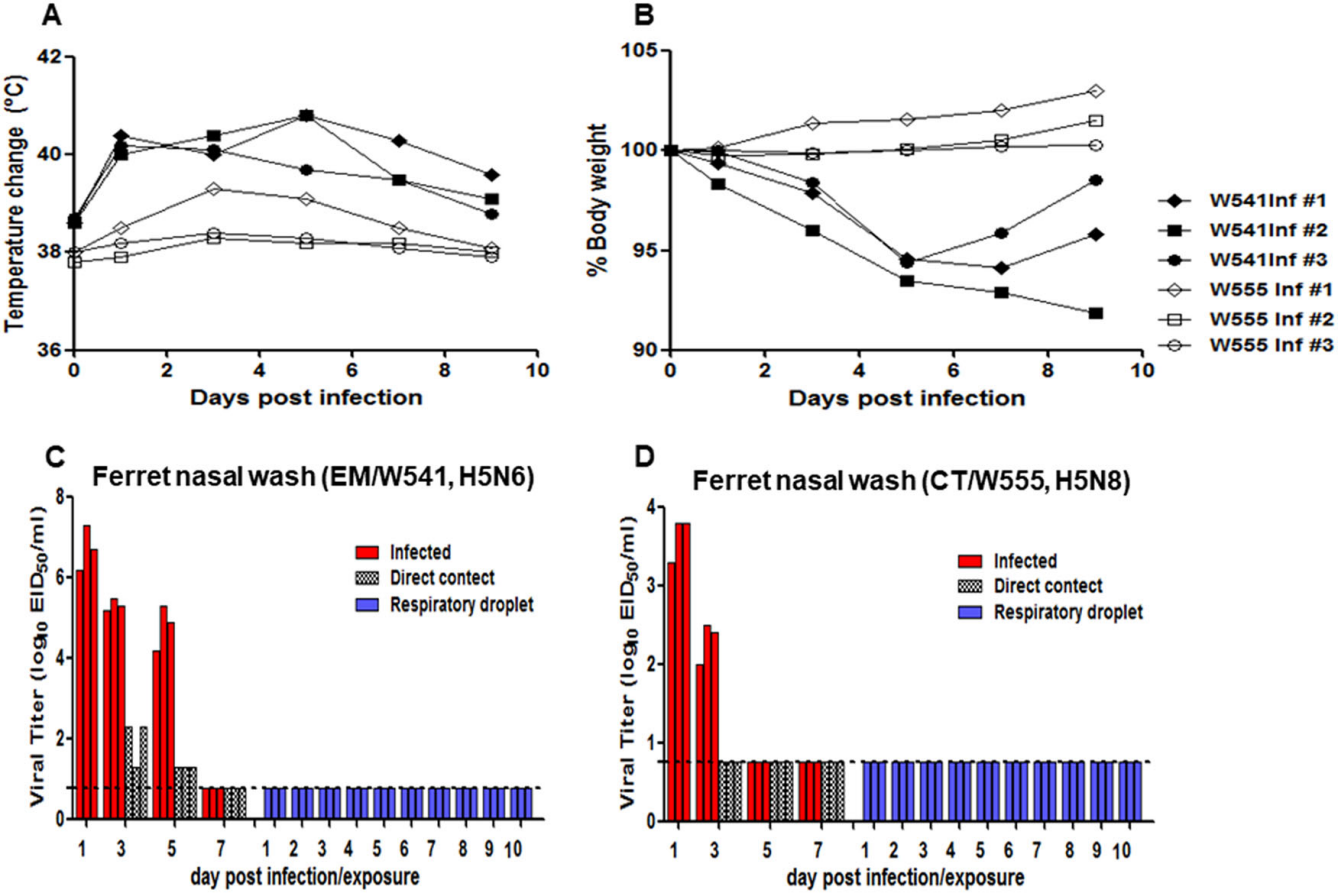
Replication of Em/W541(H5N6) and CT/W555(H5N8) in ferrets experimentally inoculated via the intranasal route with 10^6^ EID_50_/ml of each virus. Rectal temperatures and body weights were measured at 1, 3, 5, 7, 9, 11, and 14 dpi (**a** and **b**). Individual nasal wash titers are shown for ferrets inoculated with Em/W541(H5N6) and CT/W555(H5N8). To examine transmission, the inoculated animals were individually paired with a direct contact (DC) and a respiratory droplet (RD)-contact animal (1:1:1 setup, triplicate) (**c** and **d**). The mean viral titers (log_10_ EID_50_/ml) are shown for each group of mammals. The limit of virus detection was 0.7 log_10_ EID_50_/ml

To investigate the replication of Em/W541 (H5N6) and CT/W555 (H5N8) in ferrets, the established mammalian surrogate for humans in influenza research, groups of influenza-naïve ferrets (*n* = 9) were intranasally inoculated with 10^6^ EID_50_/ml of virus, and clinical signs of infection and nasal virus shedding were monitored for 14 days. Em/W541 (H5N6)-infected ferrets showed obvious clinical signs of infection (wheezing and coughing) and lost >5% of their body weight in addition to exhibiting transient elevation of body temperature (Figs. 5a, b). Virus titers in nasal wash samples averaged 5.3 and 4.8 log_10_ EID_50_/ml at 3 and 5 dpi, respectively, and were not detected by 7 dpi (Fig. 5c). Lung virus titers peaked at 6.0 EID_50_/ml by 3 dpi. Virus was detected in brain and spleen with peaks of 3.2 and 2.0 EID_50_/ml, respectively, until 5 dpi (Table 2). In contrast to Em/W541 (H5N6), CT/W555 (H5N8) infection caused mild lethargy, a moderate increase in body temperature (1.5 °C above baseline), minimal weight loss (1.0%), and no overt respiratory symptoms (Fig. 5a, b). Viral titers in nasal washes (2.0–3.8 log_10_ EID_50_/ml) were generally lower than those of Em/W541 (H5N6)-infected ferrets (Fig. 5d). Lung viral titers ranged from 3.0 to 3.5 log_10_ EID_50_/ml and virus was detected in the brain, liver, and colon, albeit at low titers (≤10^1.5^EID_50_/ml) (Table 2).

Evaluating the capacity for transmission of emerging influenza viruses is a key component of public health risk assessment^25^. To determine the transmissibility of Em/W541 (H5N6) and CT/W555 (H5N8) in ferrets, three naïve ferrets were individually co-housed, direct contact (DC), with infected ferrets at 1 dpi (Fig. 5). An additional three naïve ferrets were individually placed in the same isolator, but separated from the inoculated and DC animals by a stainless steel grill divider, to measure transmission by respiratory droplets (RD). While no virus was detected in RD contact ferrets (Fig. 5c, d), Em/W541 (H5N6) was found in nasal wash samples of DC animals at 1 and 3 dpc, and persisted up to 5 dpi. Two of the three Em/W541 (H5N6) DC ferrets seroconverted (80 HI units) as measured by HI assay (Table 3). No CT/W555 (H5N8) DC or RD ferrets exhibited signs of infection, and no virus was detected in nasal washes.

**Table 3:** Seroconversion in inoculated and contact ferrets

Virus	Exposure	Nasal wash titers (log_10_ EID_50_/ml)							Seroconversion (HI titer)
		1dpi	3dpi	5dpi	7dpi	9dpi	11dpi	14dpi	
Em/W541 (H5N6)	Inoculated	6.2	5.2	4.2	–	–	–	–	320
	Direct contact	1.3	1.3	-	–	–	–	–	<10
	RD contact	–	–	–	–	–	–	–	<10
	Inoculated	7.3	5.5	5.3	–	–	–	–	640
	Direct contact	2.3	1.3	1.3	–	–	–	–	80
	RD contact	–	–	–	–	–	–	–	<10
	Inoculated	6.7	5.3	4.9	–	–	–	–	320
	Direct contact	2.3	1.3	1.3	–	–	–	–	80
	RD contact	–	–	–	–	–	–	–	<10
CT/W555 (H5N8)	Inoculated	3.3	2.0		–	–	–	–	80
	Direct contact	–	–	–	–	–	–	–	<10
	RD contact	–	–	–	–	–	–	–	<10
	Inoculated	3.8	2.5		–	–	–	–	160
	Direct contact	-	-	-	-	-	–	–	<10
	RD contact	–	–	–	–	–	–	–	<10
	Inoculated	3.8	2.4		–	–	–	–	80
	Direct contact	–	–	–	–	–	–	–	<10
	RD contact	–	–	–	–	–	–	–	<10

Although moderate histopathologic lesions were observed in both Em/W541 (H5N6) and CT/W555 (H5N8)-infected ferret tissues, a broader range and larger number of positive cells were detected in the lungs of Em/W541 (H5N6)-infected ferrets. Further, we detected antigen-positive cells in brain and spleen from Em/W541 (H5N6)-infected animals at 3 and 5 dpi, but not in H5N8-infected ferrets (Supplementary Fig. S4).

Taken together, Em/W541 (H5N6) replicated to higher titers in the upper respiratory tract (especially in nasal washes) than CT/W555 (H5N8) and could transmit to direct-contact naïve ferrets (Fig. 5).

### Receptor-binding preference of Em/W541 (H5N6) and CT/W555 (H5N8)

Since the receptor-binding specificity of influenza virus can determine species preference, we elucidated the receptor binding patterns of these viruses by performing a solid-phase direct binding assay using the polyacrylamide (PAA)-biotin-conjugated glycans Neu5Acα2–3Galβ1–4Glc β1 (α2,3’-SL-PAA-biotin) and Neu5Acα2–6Galβ1–4Glc (α2,6’-SL-PAA-biotin)^26^. CA/07 (H1N1), which selectively binds α2,6-linked sialic acids (α2,6-SAs), was used as a control (Supplementary Fig. S1). Em/W541 (H5N6) and CT/W555 (H5N8) both had highest affinity for the avian-like SAα2,3’-SL-PAA-biotin receptor (Supplementary Fig. S1). We further analyzed the receptor-binding preference of an additional H5N6 virus, A/Environment/W558/2017 (Em/W558 (H5N6)), which was representative of viruses detected during the later stage the winter season. In agreement with the Em/W541 (H5N6) results, Em/W558 (H5N6) also showed high α-2,3 SA binding affinity and low α-2,6 SA binding affinity (Supplementary Fig. S1B). Thus, the H5N6 and H5N8 viruses have strong α-2,3 SA receptor specificity with low α-2,6 SA affinity indicating that avian species are more susceptible to this virus.

## Discussion

Aided by wild migratory birds, HPAI H5Nx viruses have spread to many Eurasian countries and even to North America; hence options to control their further dissemination are somewhat limited. Thus, timely surveillance and characterization of recent viruses in domestic poultry as well as wild birds is the best approach available to minimize the potential for human infection since domestic poultry could be a global reservoir with greater interaction with humans than wild birds. In this study, we investigated the pathobiological properties of the H5N6 and H5N8 viruses that caused a huge poultry outbreak in South Korea during the 2016/17 winter season.

The H5N6 virus exhibited high pathogenicity in avian hosts, including chickens where it had 100% mortality, a 2-day mean survival time, and broad tissue dissemination. Further, while H5N6 and H5N8 viruses replicated in all tested organs in chickens, only H5N6 robustly killed infected birds. Molecular analysis revealed one significant difference between these viruses, an NA stalk deletion present in the H5N6 virus^9^. Deletion of the NA stalk region is a major virulence determinant in chickens that was also observed in the HPAI H5N1 virus^27^. In addition, the NS1 genes of the H5N6 viruses have an 80 to 84 residue deletion compared with the H5N8 virus. A previous study demonstrated that this deletion is associated with increased pathogenicity in poultry infections with H9N2 viruses^28^. While these NA and NS1 deletions are likely contributors, further studies will be required to identify the molecular markers that distinguish H5N6 and H5N8 virulence in birds.

Of note, Em/W541 (H5N6) showed more potential to infect mammalian hosts than the H5N8 virus as inferred from viral replication in human *ex vivo* lung tissue and infection of ferrets. This result is consistent with the identification of human infections in China with H5N6 but not H5N8 viruses. Interestingly, studies have demonstrated that the 133 HA deletion seen in Em/W541 (H5N6) is associated with an alteration of the HA receptor binding pocket in H5N1 viruses from humans in Egypt that increases binding to the α-2,6 linked receptor^18,19^. A similar deletion has occurred and is maintained in viruses isolated from humans in Yunnan Changsha in China^17,19^. Analysis of publically available sequence data revealed that this deletion is overrepresented in recent human H5N6 viruses (70% vs 3.1% in human and avian isolates, respectively) implying an association of this change with elevated zoonotic risk (Supplementary Table S2). Although the receptor-binding specificity of the Korean H5N6 viruses still showed a preference for the α-2,3 sialic acid avian receptor, the virus could replicate in the upper respiratory tract of ferrets at a rate much higher than the H5N8 virus (peak titers, 6.0 VS 2.5 log_10_EID_50_/ml in H5N6 and H5N8, respectively) as well as in human NHBE cells and ex vivo lung tissues. Supporting an elevated zoonotic risk of Em/W541 (H5N6) over CT/W555 (H5N8) was the finding that only the former virus was transmitted to direct contact ferrets where it was shed for 3 to 5 days; a property not shared with previously circulating HPAI H5N1 and H5N8 viruses in Korea^13,29,30^. This phenomenon was previously reported in China^31^ where only H5N6 caused human infection^32–34^. However, in our study direct contact ferrets showed markedly attenuated virus titers in nasal swabs and clinical symptoms compared with H5N6 directly infected ferrets (Fig. 5). Therefore, further studies are needed to understand the significance of direct transmission in ferrets for mammalian host adaptation.

While the H5N6 and H5N8 viruses had similar MLD_50_ values, the H5N6 virus spread more systematically in both mice and ferrets and was detected in brain, spleen, heart, kidney and/or liver. Viral dissemination is a critical characteristic of virulence and can cause multi-organ dysfunction, which is the major cause of death in HPAI H5N1-infected humans^35^. In mammals, a cytokine storm, characterized by the deregulated and excessive production of inflammatory cytokines, correlates with increased morbidity and mortality during multiple pathogenic respiratory viral infections^36^. Although the H5N6 and H5N8 viruses were less pathogenic than the HPAI H5N1 virus, both viruses are pathogenic enough to cause upregulation of pro-inflammatory cytokines and mortality in infected mice (Fig. 3). Our data are consistent with previous findings, including those of Kim et al. showing that the H5N8 viruses are strong inducers of the proinflammatory cytokine response, which is consistent with its pathogenicity^13^.

Since the first H5N1 virus outbreak in 2003, six HPAI virus outbreaks have occurred in South Korea. However, the spread of these viruses in the field was quickly controlled as a result of the rapid diagnosis of the infections due to the high pathogenicity of these viruses in poultry. In contrast, clade 2.3.4 H5Nx viruses are usually mild in ducks leading to delayed diagnosis of infections and persistently spread in the wild, such as occurred during an outbreak which started in 2014. In order to overcome these difficulties, it is necessary to both educate farmers and to actively collect routine samples. Further, continuous surveillance monitoring is essential to understand the geographical distribution of HPAI viruses and the international exchange information on the emergence of new variant viruses is also necessary.

Despite the large number of H5N6 human infections with more than 30% mortality in China, there is not yet direct evidence of human to human transmission (http://www.who.int/csr/don/07-december-2016-ah5n6-china/en/). However, with large outbreaks in South Korea and global spread of H5N8 viruses, there is the potential for increasing genetic evolution of the H5Nx viruses with unpredictable consequences for zoonotic and pandemic risk. Thus, this study re-emphasizes the importance of active and continuous surveillance of avian influenza viruses in wild birds and poultry in concert with the characterization of virulence in mammalian hosts.

## Materials and methods

### Cells

Madin–Darby canine kidney (MDCK) cells (American Type Culture Collection, Manassas, VA, USA) were grown and maintained in Eagle’s minimum essential medium with Earle’s salts (Lonza, Basel, Switzerland) containing 5% fetal bovine serum (Gibco Life Technologies, Grand Island, NY, USA). Primary normal human bronchial epithelial (NHBE) cells were purchased from ScienCell Research Laboratories (Carlsbad, CA, USA) and differentiated as previously described^37^. All cells were incubated at 37 °C in 5% CO_2_ until use. Upon isolation, tissues were minced, trypsinized and cultured at 37 °C in 5% CO_2_ in Dulbecco’s modified Eagle’s medium containing 10% fetal bovine serum, 2 mM glutamine and antibiotics. Cell viability was assessed via Trypan blue exclusion assays and was not less than 90% for any preparation.

### Viral propagation

Except for A/California/07/2009(H1N1) (CA/07(H1N1)), which was propagated in MDCK cells, the HPAI H5 viruses [A/Environment/Korea/W541/2016 (Em/W541(H5N6), and A/Common Teal/Korea/W555/2017 (CT/W555(H5N8)] were isolated from wild bird fecal samples during the 2016 and 2017 winter seasons and were grown in specific pathogen free (SPF), 10-day-old embryonated chicken eggs. Supernatants (allantoic fluids and cell culture) were harvested at 48 h post-infection (hpi), aliquoted into cryovials (1 ml each), and stored at −80 °C until use. Stock viral titers were determined by 50% egg infectious dose (EID_50_) and 50% tissue culture infectious dose (TCID_50_) end-point titrations. All experiments with HPAI H5 including virus titrations in biological samples and receptor-binding assays, were conducted in an enhanced biosafety level 3 (BSL3) containment facility.

### Molecular analysis

Virus sequences were prepared and analyzed as previously described^38^. Briefly, gene sequences of Em/W541(H5N6) and CT/W555(H5N8) were obtained by Cosmo Genetech (Seoul, Korea) using an ABI 3730XL DNA sequencer (Applied Biosystems, Foster City, CA, USA). Sequences were analyzed and compiled with DNAStar 5.0 (DNAStar, Madison, WI, USA). Closely related viruses were identified by basic local alignment search tool analysis.

### Virus titrations

Virus titers in virus stocks, oropharyngeal and cloacal swabs, nasal washes, homogenized/clarified organ tissue samples, and culture supernatants were determined by performing endpoint titrations in 10-day-old embryonated chicken eggs, monolayers of MDCK cells, or both. Eggs or MDCK cells were inoculated with 10-fold serial dilutions of each sample in phosphate-buffered saline solution or fetal bovine serum-free media containing antibiotics. After a 48 h incubation at 35 °C, the presence of viruses was detected by a standard HA assay using 0.5% turkey erythrocytes. Mean virus titers are expressed as log_10_EID_50_ or as log_10_TCID_50_ per unit-sample (ml or g) tested. The limit of virus detection was set at 0.75 or 2.5 log_10_ per unit-sample tested.

### Growth kinetics of virus in vitro and ex vivo

MDCK and differentiated primary NHBE cells were infected in triplicate in six-well plates with Em/W541(H5N6), CT/W555(H5N8), and CA/07(H1N1) at multiplicities of infection of 0.001, 0.1 or 0.01. After incubation at 35.6 °C for 45 min to 1 h, the viral inoculation supernatants were replaced with serum-free medium appropriate for each cell line. Viral growth rates in all cells were determined three times in duplicate at 35.6 °C in the absence of L-1-tosylamido-2-phenylethyl chloromethylketone-treated trypsin. Cell culture supernatants were harvested at 6, 12, 24, 48, and 72 hpi for virus titration in MDCK cells, as specified above.

Fresh human lung tissue fragments were surgically obtained ex vivo, from patients undergoing removal of respiratory tissues for screening for possible carcinoma. Only normal, non-malignant tissue fragments that were not needed for clinical diagnosis were used for infection, as previously described^13^. Briefly, excised tissues were cubed (5 mm, 34 mm, 32 mm), rinsed and incubated in Roswell Park Memorial Institute 1640 medium (Gibco) with L-glutamine and antibiotics for 2–3 h at 35.6 °C in 5% CO_2_. Em/W541 (H5N6), CT/W555 (H5N8), and CA/07 (H1N1) (10^6^ log_10_EID_50_/ml) were used to infect tissue sections (triplicates of 25 fragments per virus) in six-well plates. After 1 h of virus adsorption at 35 °C, the infected tissue sections were rinsed and segregated (five blocks each) in 12-well cell culture vessels containing 1 ml of Roswell Park Memorial Institute 1640 medium supplemented with 0.5 mg/ml L-1-tosylamido-2-phenylethyl chloromethylketone-treated trypsin solution (Sigma-Aldrich, St Louis, MO, USA), 1% bovine serum albumin and antibiotics. At 12, 24, 48, and 72 hpi, lung tissue sections were homogenized, and the homogenates were clarified by centrifugation. The titers of the viruses in tissue homogenates and cell culture supernatants were determined by EID_50_ assays.

### Neuraminidase inhibitor (NAI) resistance detection test

NA inhibitors oseltamivir carboxylate and zanamivir were purchased from Toronto Research Chemicals, Inc. (Canada), and peramivir was acquired from Green Cross (Korea). The chemiluminescent NA inhibitor assay was conducted using the commercially available NA-Star kit (Applied Biosystems, Foster City, CA). NA-Star substrate was used at a final concentration of 100 μM. Briefly, 25 μl of half-log dilutions of neuraminidase inhibitors (0.03–1000 nM) in NA-Star Assay buffer were added to each well of a white 96-well microplate plate, then 25 μl of virus dilution was added and plates were pre-incubated at 37 °C for 20 min. Diluted NA-Star (0.01 mM; 10 µL) was then added to each well and incubated for 10 min at room temperature. Finally, 60 μl of NA-Star Accelerator was added to each well, and the luminescence at 450 nm wavelength was read immediately (1.0 s/well). The 50%–inhibitory concentration (IC_50_) values were determined by using GraphPad Prism^TM^ software (v5) for non-linear regression analysis.

### Experimental infection of chickens and ducks

To calculate the 50% chicken and duck lethal dose (Ck LD_50_ and Dk LD_50_) of Em/W541(H5N6) and CT/W555(H5N8), five-week-old specific-pathogen-free (SPF) white leg-horn chickens (*G. gallus domesticus*, CAVac Lab. Co., Ltd., Daejeon, Korea) (*n* = 6/group) and 4-week-old influenza seronegative domestic ducks (*Anas platyrhynchos domesticus*) (*n* = 6/group) were inoculated with 10-fold serial diluted viruses ranging from 10^0^ to 10^6^ EID_50_ and monitored for 14 days. Additional groups of 21 chickens and ducks were oronasally (o.n.) inoculated with 10^6^ EID_50_/ml of each virus to examine virus pathogenesis (*n* = 12/group) and transmission (*n* = 9/group). Oropharyngeal and cloacal swab samples were collected from chickens (1, 3, and 5 dpi) and ducks (1, 3, 5, and 7 dpi). Lung, brain, kidney, spleen, intestine, heart, and cloaca were collected from three animals each using individual sterile equipment to determine virus tissue distribution. Tissue samples were homogenized in 1 × PBS containing antibiotics and wereclarified by centrifugation. Virus titrations were performed as described above. To test for virus transmission, six contact birds were cohoused with the three infected hosts starting at 1 dpi and were observed daily. Tracheal and cloacal swabs were collected from the inoculated animals on alternate days and from contact birds every day.

### Experimental infection of mice and ferrets

To determine 50% mouse lethal doses (MLD_50_) of Em/W541(H5N6) and CT/W555(H5N8), groups of 6 female five-week-old BALB/c mice (Samtaco, Seoul, Korea) were intranasally (i.n.) inoculated with 30 ul of 10-fold serially diluted viruses ranging from 10^0^ to 10^6^ EID_50_ and monitored for 14 days. Additional groups of 20 mice were i.n. inoculated with 30 ul of 10^5^ EID_50_/ml of each virus to examine their growth kinetics in mouse lungs. Five mice per group were sacrificed at 1, 3, 5, and 7 dpi for various tissue collections. Virulence levels in mice were categorized as low (MLD_50_ > 6.5 log_10_ EID_50_), medium (3 log_10_ EID_50_ < MLD_50_ ≤ 6.5 log_10_ EID_50_), or high (MLD_50_ ≤ 3 log_10_EID_50_) pathogenicity.

Groups of three female 15-week-old to 18-week-old ferrets (*Mustella putorios furo*) (I.D bio corporation, Republic of Korea) seronegative for influenza virus antibodies were intranasally and intratracheally inoculated with 10^6^ EID_50_/ml of each of Em/W541(H5N6) or CT/W555(H5N8) virus in 1 ml of sterile PBS (divided between two plastic syringes for separate inoculation of each nostril) under Zoletil/xylazine mixture anesthesia (Zoletil 50^®^, 80 mg/kg, Virbac, France; Rompun^®^, 20 mg/kg, Bayer HealthCare, Germany). At 1 dpi, the three inoculated animals were individually paired for cohousing with a direct contact (DC) ferret and a respiratory-droplet (RD) contact ferret. The infected and RD contact ferrets were kept 5 cm apart by a wire frame cage, preventing direct contact between animals but allowing the spread of the influenza virus via aerosol droplets. The body weight and temperature of the animals were monitored every other day. Nasal washes were collected from the infected ferrets every other day for 9 days beginning at 1 dpi and daily from contact ferrets starting at 1 day post-exposure. Virus titrations of nasal washes and various tissue organs were determined by EID_50_ assays.

### Immunostaining

Tissue samples were fixed in formalin for 24 h before embedding in paraffin. One micrometer sections were prepared by Leica RM2235 microtome. The antigen retrieval were performed by placing sections in 0.1% trypsin for 15 minutes at 37 ˚C. The tissue section were incubated with 1/50 dilution of primary monoclonal antibody against type A Influenza nucleoprotein (NP) (AbDSerotec, Kidlington, Oxford, UK). The immunohistochemistry staining to detect Influenza A viral nucleoprotein were done using commercial Vectastain ABC Elite HPR kit (Vector Laboratories, Burlingame, CA, USA) following the procedures recommended by manufacture.

### Receptor binding assays

Receptor affinity was determined in a solid-phase direct virus-binding assay as previously described. In brief, influenza viruses were bound to fetuin-coated plates at 4 ˚C overnight. Biotinylated glycans (a2,3’SL; a2,6’SL; or a2,6’SLN; Glycotech Corporation, Gaithersburg, MD, USA) were added to influenza-coated plates at varying dilutions and incubated for 4 h. Glycan binding was detected by adding horseradish peroxidase-conjugated streptavidin (Invitrogen, Carlsbad, CA, USA) followed by 3,3′,5,5′-tetramethylbenzidine substrate (Sigma-Aldrich, St Louis, MO, USA); the resulting absorbance at 450 nm was measured in a VICTOR3 1420 multilabel-counter plate reader (Perkin-Elmer, Waltham, MA, USA).

### Cytokine and chemokine measurement

Broncho alveolar lavage fluid (BALF) samples were collected from mouse lungs at 1, 3, 5, 7, and 9 dpi or from uninfected control mouse lungs (5 mice/group). The BALF samples were centrifuged at 12,000 r.p.m. for 5 min at 4 °C, aliquoted, and stored at −70 °C until analysis. A multiplex biometric immunoassay using fluorescently dyed microspheres conjugated with monoclonal antibodies specific for target proteins was used for cytokine measurements according to the manufacturer’s instructions (Bio-Plex Pro™ Mouse Cytokine Th1/Th2 Assay). Levels of the following cytokines were measured: IL-2, IL-4, IL-5, IL-10, IL-12p70, interferon-gamma (INF-γ), tumor necrosis factor-alpha (TNF-α), and chemokine (GM-CSF). Briefly, 20 μl of BALF was diluted 1:4 and incubated with antibody-coupled beads. Complexes were washed, incubated with a biotinylated detection antibody and labeled with streptavidin–phycoerythrin before cytokine concentrations were measured. A range of 5000–25,000 pg/ml of recombinant cytokines was used to establish standard curves and to maximize the sensitivity and dynamic range of the assay. The cytokine levels were determined using a Luminex™ Instrumentation Systems multiplex array reader (Bio-Plex Workstation from Bio-Rad Laboratories). Cytokine concentrations were calculated using software provided by the manufacturer (Bio-Plex Manager Software).

### Ethics statement

All animal experimental protocols performed in this study strictly followed general animal care guidelines mandated under the Guidelines for Animal Use and Care of the Korea Center for Disease Control (KCDC). They were approved by the Laboratory Animal Research Center (approval No CBNUR-1041–16), which is a member of the Institutional Animal Care and Use Committee of Chungbuk National University.

Animal experiments were progressed in an enhanced animal biosafety level 3 facility (BSL3) at Chungbuk National University permitted by the K-CDC (permit number KCDC-14-3-07). Ex vivo experiments involving human respiratory tissues were conducted using protocols approved by the Ethics Committee of the Faculty of Medicine at Chungbuk National University in Cheongju, Korea (IRB-2017-05-002). The experimental protocol was comprehensively explained to patients undergoing tissue biopsies and duly signed written consent forms were obtained.

### Statistical analysis

The differences among log10-transformed viral titers of different viruses were compared using Student’s *t*-test or one-way analysis of variance. The statistical analyses were carried out using GraphPad Prism^TM^ software (v5) (La Jolla, CA, USA).

## Electronic supplementary material

Supplementary Table 1

Supplementary Table 2

Supplementary Table 3

Supplementary Figure 1

Supplementary Figure 2

Supplementary Figure 3

Supplementary Figure 4
