# Supplementary material for: Comparison of the pathogenic potential of highly pathogenic avian influenza (HPAI) H5N6, and H5N8 viruses isolated in South Korea during the 2016–2017 winter season
Source: Emerg Microbes Infect. 2018 Mar 14;7:29. doi: 10.1038/s41426-018-0029-x (PMC5849756; doi:10.1038/s41426-018-0029-x)
Supplement: Supplementary file 1 — Supplementary Table 1 [file 41426_2018_29_MOESM1_ESM.docx]

Supplementary Table S1. Molecular comparison of influenza A subtype H5 viruses emerging from 2016/17 with previous Korean viruses and similar isolates.

| Viruses^†^ | HA clade | HA sequence (aa) | | | | | | | | | HA  deletion | NA stalk deletion | NS1 | | | PB2  sequence at aa | | Expression of PB1-F2 protein |
| --- | --- | --- | --- | --- | --- | --- | --- | --- | --- | --- | --- | --- | --- | --- | --- | --- | --- | --- |
|  |  | Cleavage site | Receptor binding sites | | | | | | | |  |  | Deletion of aa 80‑84 | Aa residue at | |  |  |  |
|  |  | 335-348 | 158 | 193 | 222 | 224 | 226 | 227 | 228 | 318 | 133 | 49-68 |  | 92 | C-term | 627 | 701 |  |
| **Em/Korea/W541/16** | 2.3.4.4 | RERRR_KR/G | N | N | Q | N | Q | Q | G | T | YES | YES | YES | E | ESEV | E | D | YES |
| **CT/Korea/W555/17** | 2.3.4.4 | REKRR_KR/G | N | N | Q | N | Q | R | G | T | NO | NO | NO | D | GSEV | E | D | YES |
| Yunnan/China/0127/15 | 2.3.4.4 | RERRR_KR/G | N | N | Q | N | Q | R | G | T | YES | YES | NO | D | KPEV | K | D | YES |
| Changsha/China/1/14 | 2.3.4.4 | RERRR_KR/G | N | N | Q | N | Q | R | G | T | YES | YES | YES | E | ESEV | E | D | YES |
| MDk/Korea/W452/14 | 2.3.4.6 | RERRR_KR/G | N | N | Q | N | Q | R | G | T | NO | NO | NO | D | ESEV | E | D | YES |
| BDk/Korea/Gochang1/14 | 2.3.4.6 | RERRR_KR/G | N | N | Q | N | Q | R | G | T | NO | NO | NO | D | ESEV | E | D | YES |
| Egypt/MOH/7271/14 | 2.2.1.2 | GERRRKKR/G | N | R | K | N | Q | S | G | T | YES | YES | YES | D | ESEV | K | D | YES |

Aa: amino acid; BDk: Breeder duck; C-term: 4 amino acid sequence at the C-terminal end; Em: environment; HA: haemagglutinin; HPAI: highly pathogenic avian influenza; MDk: mallard duck; RBS: receptor binding site; MOH: Ministry of health; NA: neuraminidase; NS: nonstructural protein; PB: polymerase basic protein.

^†^ The isolates in boldface are the 2016/2017 Korean HPAI H5N6 and H5N8 viruses examined in this study.
