# Supplementary material for: Comparison of the pathogenic potential of highly pathogenic avian influenza (HPAI) H5N6, and H5N8 viruses isolated in South Korea during the 2016–2017 winter season
Source: Emerg Microbes Infect. 2018 Mar 14;7:29. doi: 10.1038/s41426-018-0029-x (PMC5849756; doi:10.1038/s41426-018-0029-x)
Supplement: Supplementary file 2 — Supplementary Table 2 [file 41426_2018_29_MOESM2_ESM.docx]

Supplementary Table S2. Single nucleotide polymorphism (SNP) analysis of H5 viruses.

| **Protein** | **Amino acid  change^a^** | **Polymorphism(s), iNdatabase(freequncy [%]),c** | | |  |
| --- | --- | --- | --- | --- | --- |
|  |  | **Avian** | **# Sequences** | **Human** | **# Sequences** |
| H5Nx(HA) | 133 | L(96.5). S(2.7), Del(0.9) | 452 | Del(70), S(30) | 10 |
|  | 227 | R(61.7), S(37.8), G(0.5) | 452 | R(70), S(30) | 10 |
| H5N2(HA) | 133 | L(100) | 85 | NA | NA |
|  | 227 | S(80.0), R(18.8), G(1.2) | 85 | NA | NA |
| H5N5(HA) | 133 | S(100) | 10 | NA | NA |
|  | 227 | R(100) | 10 | NA | NA |
| H5N6(HA) | 133 | L(89.1), S(7.8), Del(3.1) | 129 | Del(70), S(30) | 10 |
|  | 227 | R(92.2), S(7.8) | 129 | R(70), S(30) | 10 |
| H5N8(HA) | 133 | L(100) | 237 | NA | NA |
|  | 227 | R(56.5), S(43.0), G(0.4) | 237 | NA | NA |

^a^H5 numbering.
