# Supplementary material for: Comparison of the pathogenic potential of highly pathogenic avian influenza (HPAI) H5N6, and H5N8 viruses isolated in South Korea during the 2016–2017 winter season
Source: Emerg Microbes Infect. 2018 Mar 14;7:29. doi: 10.1038/s41426-018-0029-x (PMC5849756; doi:10.1038/s41426-018-0029-x)
Supplement: Supplementary file 3 — Supplementary Table 3 [file 41426_2018_29_MOESM3_ESM.docx]

Supplementary Table S3. Virus sensitivity to NA inhibitor compounds *in vitro*.

| **Compound** | **IC_50_ value^a^** | | | |
| --- | --- | --- | --- | --- |
|  | **Em/W541(H5N6)** | **CT/W555(H5N8)** | **MDk/W452(H5N8)** | **CA/04(H1N1)_H274Y_^c^** |
| **Oseltamivir carboxylate^b^** | 6.62 | 5.83 | 12.25 | 2565 |
| **Peramivir** | 1.93 | 0.86 | 0.91 | 252.4 |
| **Zanamivir** | 9.64 | 2.06 | 2.47 | 3.83 |
| **Laninamivir** | 11.27 | 1.05 | 1.19 | 4.07 |

^a^IC_50_ value: mean nM of triplicate reactions.

^b^Oseltamivir carboxylate is the active form of oseltamivir.

^c^The 2009 pandemic virus CA/04(H1N1), A/Califormia/04/2009(H1N1), was modified to contain the H274Y resistance marker in its NA gene, which served as a positive control against the NA inhibitor compounds tested.
