# Supplementary figures and images for: Comparison of the pathogenic potential of highly pathogenic avian influenza (HPAI) H5N6, and H5N8 viruses isolated in South Korea during the 2016–2017 winter season
Source: Emerg Microbes Infect. 2018 Mar 14;7:29. doi: 10.1038/s41426-018-0029-x (PMC5849756; doi:10.1038/s41426-018-0029-x)

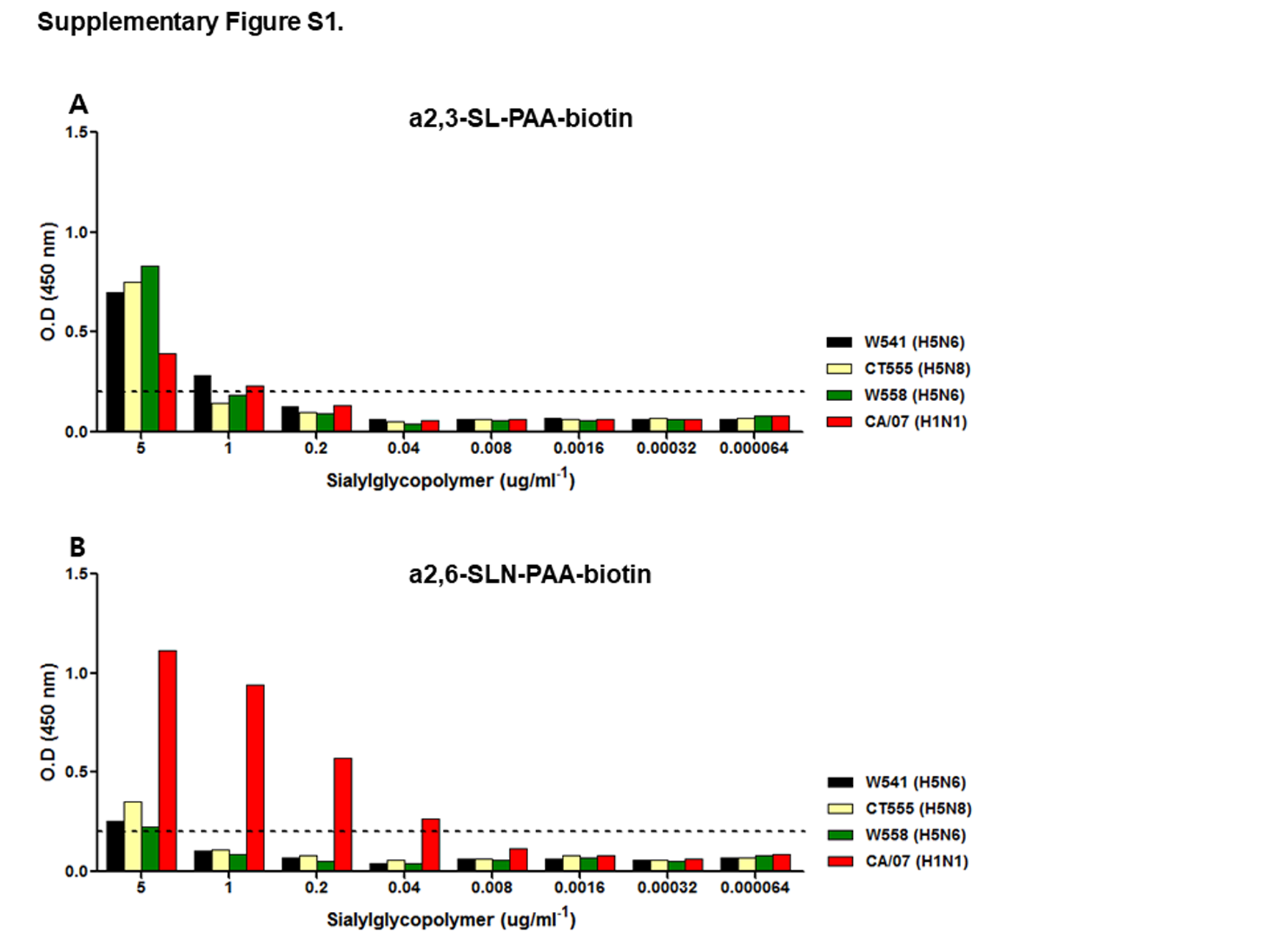

Supplement: Supplementary file 4 — Supplementary Figure 1 [file 41426_2018_29_MOESM4_ESM.tif]

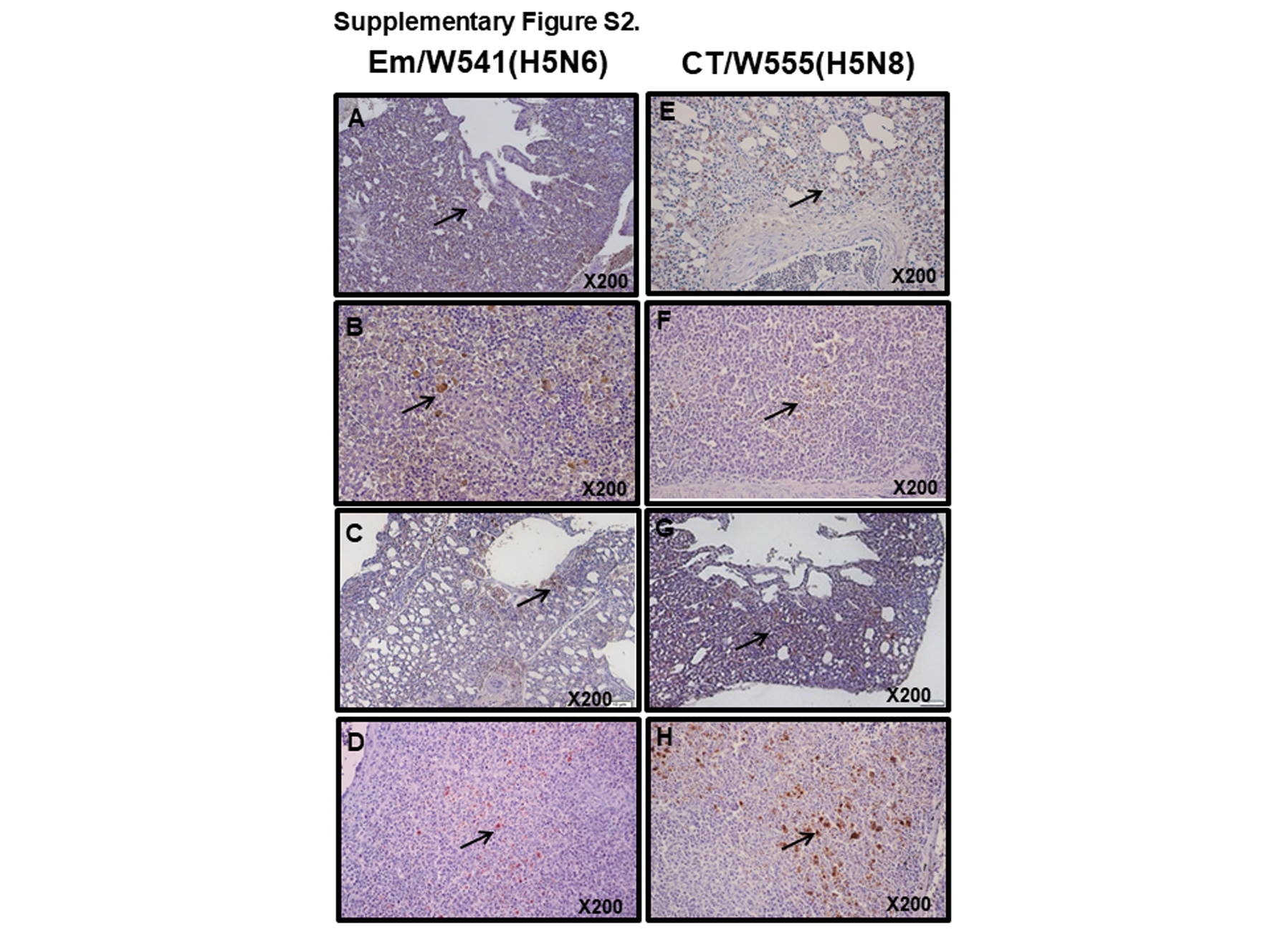

Supplement: Supplementary file 5 — Supplementary Figure 2 [file 41426_2018_29_MOESM5_ESM.tif]

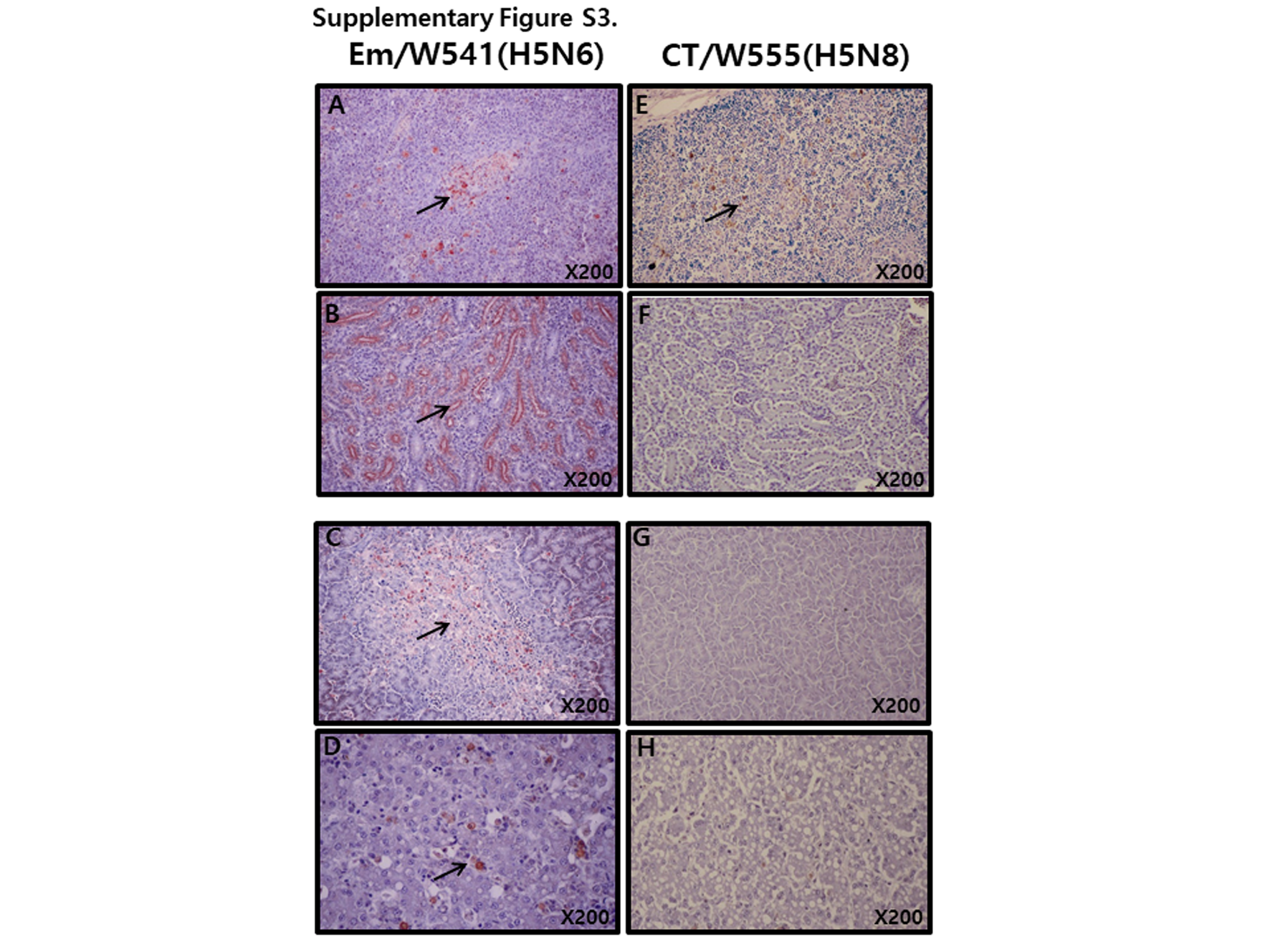

Supplement: Supplementary file 6 — Supplementary Figure 3 [file 41426_2018_29_MOESM6_ESM.tif]

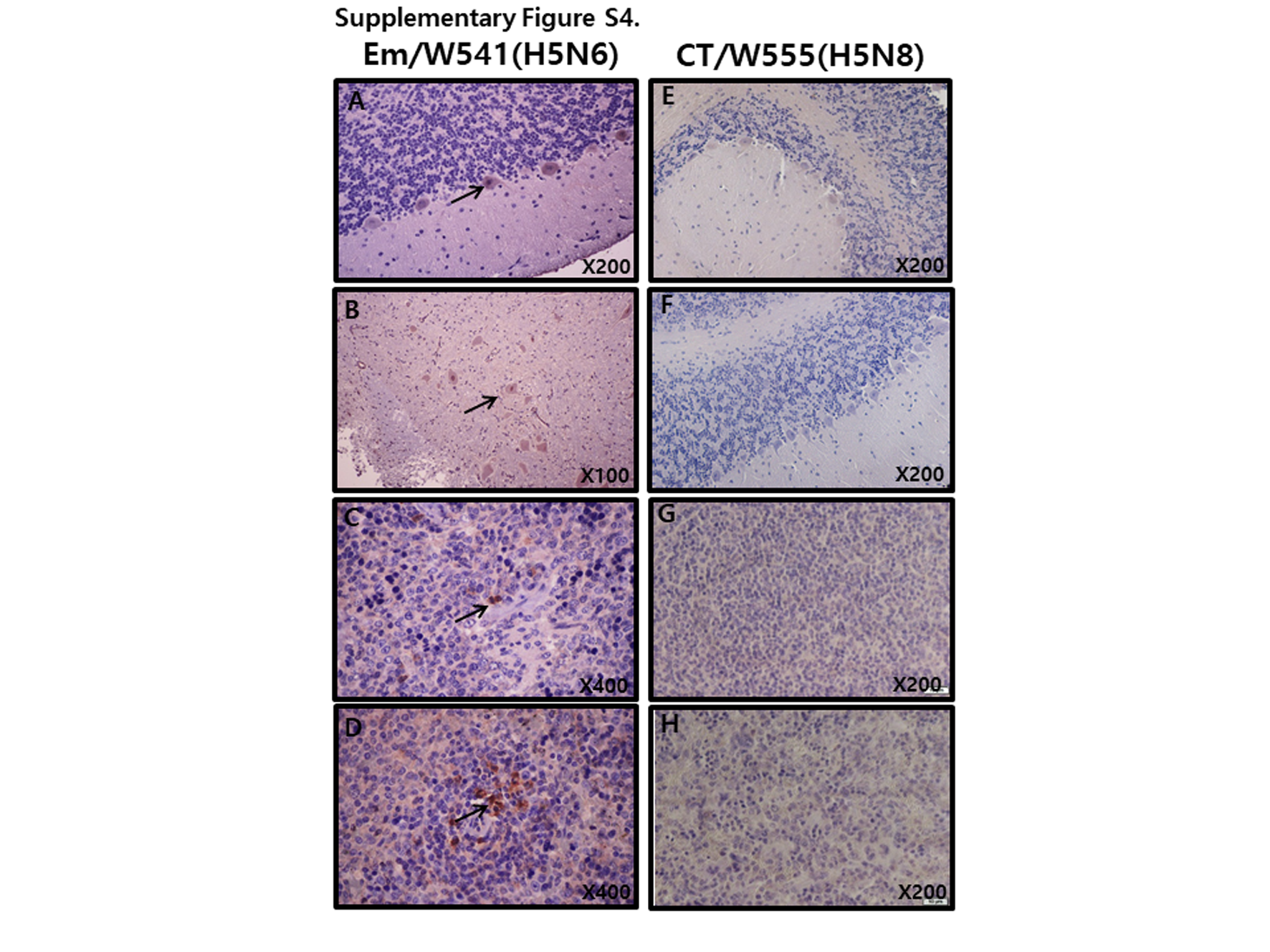

Supplement: Supplementary file 7 — Supplementary Figure 4 [file 41426_2018_29_MOESM7_ESM.tif]
